# Phytochemistry of Three Ecuadorian Lamiaceae: *Lepechinia heteromorpha* (Briq.) Epling, *Lepechinia radula* (Benth.) Epling and *Lepechinia paniculata* (Kunth) Epling

**DOI:** 10.3390/plants8010001

**Published:** 2018-12-20

**Authors:** Gianluca Gilardoni, Jorge Ramírez, Mayra Montalván, Willan Quinche, Jackeline León, Lita Benítez, Vladimir Morocho, Nixon Cumbicus, Carlo Bicchi

**Affiliations:** 1 Departamento de Química y Ciencias Exactas, Universidad Técnica Particular de Loja (UTPL), Calle M. Champagnat s/n, 1101608 Loja, Ecuador; jyramirez@utpl.edu.ec (J.R.); mayste_95@hotmail.com (M.M.); wstalin.14@gmail.com (W.Q.); jackileo@hotmail.es (J.L.); lsbenitez1@utpl.edu.ec (L.B.); svmorocho@utpl.edu.ec (V.M.); nlcumbicus@utpl.edu.ec (N.C.); 2 Dipartimento di Scienza e Tecnologia del Farmaco, Università degli Studi di Torino, Via P. Giuria 9, 10125 Torino, Italy; carlo.bicchi@unito.it

**Keywords:** Ecuador, Lamiaceae, essential oil, ledol, caryophyllene oxide, spathulenol, angustanoic acid E, 5-hydroxy-4′,7-dimethoxy flavone, guaiol, carnosol

## Abstract

In this research, the leaves of *Lepechinia heteromorpha* (Briq.) Epling, *Lepechinia radula* (Benth.) Epling and *Lepechinia paniculata* (Kunth) Epling have been collected in order to perform a phytochemical study. The first species was distilled to obtain a novel essential oil (EO), while the others were submitted to ethyl acetate extraction and secondary metabolite isolation. The chemical composition of the EO from *L. heteromorpha* has been investigated by Gas Chromatography-Mass Spectrometry (GC-MS) and Gas Chromatography with Retention Indices (GC(RI)), identifying 25 constituents. A major compound, (−)-ledol (21.2%), and a minor compound, (−)-caryophyllene oxide (1.0%), were isolated from the EO and their structures confirmed by Nuclear Magnetic Resonance (NMR) spectroscopy. Other major constituents of the EO were viridiflorene (27.3%), (*E,E*)-α-farnesene (1.4%), spirolepechinene and (*E*)-β-caryophyllene (7.1% each), *allo*-aromadendrene (6.1%), camphor (1.7%), limonene (1.3%) and β-phellandrene (4.6%). The enantiomeric composition of the EO monoterpene fraction was also studied, determining the enantiomeric excess and distribution of α-pinene, limonene, β-phellandrene and camphor. The ethyl acetate extract of *L. radula* and *L. paniculata* were fractionated by column chromatography. Spathulenol, angustanoic acid E and 5-hydroxy-4′,7-dimethoxy flavone were isolated from *L. radula* extract; ledol, guaiol and (−)-carnosol were found in *L. paniculata.*

## 1. Introduction

Ecuador is one of the seventeen mega-biodiverse countries in the world [1]. Therefore, it is a great source of new natural products, since most of the botanical species have never been studied before for their secondary metabolites [2]. Furthermore, Ecuador hosts many indigenous cultures, maintaining an ancestral knowledge that includes traditional medicine. In recent years, this cultural heritage has often been investigated to select interesting botanical targets, in particular to find new biologically active compounds. The secondary metabolites can be classified as volatile or non-volatile. If the volatile fraction is isolated by steam distillation, it is an essential oil (EO). From a qualitative point of view, EOs are often mixtures of known compounds; however, their quantitative composition can be very different from species to species. The quali-quantitative composition of an EO is responsible for its sensorial and biological properties. Furthermore, several components in an EO are chiral compounds and they are often present as pure enantiomers or in an enantiomeric excess and, seldom, in racemic form [3]. Since the enantiomers of a chiral compound may have different biological and sensorial properties [4], the recognition of chiral compounds by enantioselective GC is a useful but seldom applied step in the chemical study of a plant EO fraction [5].

Our interest in the genus *Lepechinia* is due to the abundance of EOs and the variety of secondary metabolites produced [6,7,8,9,10,11,12]; it is not based on an ethnomedical evidence, traditional use or biological activity. Therefore, this study can be considered as a contribution to the phytochemistry of genus *Lepechinia*, reporting the first chemical description of the EO distilled from *Lepechinia heteromorpha* (Briq.) Epling. Concerning *Lepechinia radula* (Benth.) Epling and *Lepechinia paniculata* (Kunth) Epling, two previous studies, describing the respective EOs, are now complemented with the purification and characterization of some major secondary metabolites, obtained by ethyl acetate extraction.

*L. heteromorpha* is botanically described with the accepted name *Sphacele conferta* Benth and with the synonym *Sphacele heteromorpha* Briq. [13]. It is a shrub native of the Andean region, growing wild between 2000 and 3000 m above the sea level. The species is distributed in Ecuador, where it has been found in the provinces of Azuay, Cañar and Loja [14]. To the best of the authors’ knowledge, no chemical or bio-activity studies about this plant have been reported in literature until now.

*L. radula* is also a shrub, native of the Andean region and growing between 2000 and 2500 m above sea level. This plant is described with the accepted name *Sphacele radula* Benth. [15] and three botanical synonyms are known: *Alguelagum cordifolium* (Benth.) Kuntze, *Alguelagum radula* (Benth.) Kuntze and *Sphacele cordifolia* Benth [16]. The plant has been described in Ecuador in the provinces of Azuay and Loja [17]. An EO composition of this species has already been reported in the literature [18].

*L. paniculata* is an endemic shrub, growing wild between 2000 and 2500 m above sea level. It has been described in the Ecuadorian provinces of Azuay and Loja. Synonyms of this species are: *Alguelagum paniculatum* (Kunth.) Kuntze, *Sideritis paniculata* Kunth, and *Sphacele paniculata* (Kunth) Benth. [19]. According to some literature, an accepted name is *Lepechinia betonicifolia* (Lam.) Epling [20], although other sources consider it as a different species [14]. An EO of this plant has already been studied [21].

To the best of the authors’ knowledge, nothing is reported in the literature about secondary metabolites from organic solvent extracts of *L. radula* and *L. paniculata*.

Concerning the traditional use of these plants, only *L. paniculata* is known with a specific name and ethnomedical use. According to the literature [22], this species is called *llanllun* or *azul yallun* by the Kichwa people of the Ecuadorian Andean region, who use it in both supernatural and physical applications. The buds are tied at the forehead for treating the “mal de aire”, a sort of evil eye. The same application is considered effective against headache, while flower infusions are used to treat nervous diseases. However, two different terms seem to be used to indicate generically that the species belongs to the whole genus *Lepechinia*: *zhalshon negro grande* in Spanish and *yana zhalshon* in Kichwa [23]. Among the indigenous people Saraguro, settled in the province of Loja, the leave are used to treat “mal de aire” and aches in muscles and bones, simply binding them to the aching area.

## 2. Results

### 2.1. EO from L. Heteromorpha Leaves

#### 2.1.1. Chemical Analysis

The EO distilled from leaves of *L. heteromorpha* was a yellowish waxy solid, easily melted at low temperatures. Due to its physical state, it was recovered dissolving it in dichloromethane and the yield of each distillation was determined through semi-quantitative Gas Chromatographic analysis with Flame Ionization Detector (GC-FID). The resulting average yield was 0.06 ± 0.029 % (*w/w*), referred to fresh plant material. The detected and quantified components cover 99.1% of the EO; its specific rotatory power was [α${]}_{D}^{20}$ = −5.6 (c 5.07 in CH_2_Cl_2_). The qualitative and semi-quantitative compositions of the EO are reported in Table 1.

The sesquiterpene fraction of an EO is often very complex and the characterization of single components through preparative isolation and spectroscopic elucidation is usually recommended. The common way is by preparative column chromatography, on normal or reversed phase, followed by NMR and MS experiments. The main problem in achieving this result is the very similar polarity of the isomeric terpenes, which usually does not permit an efficient preparative separation. In the case of our EO, only ledol and caryophyllene oxide could be separated and purified by liquid chromatography, due to the different relative polarity.

#### 2.1.2. Enantioselective GC Analysis

**Figure 1:**
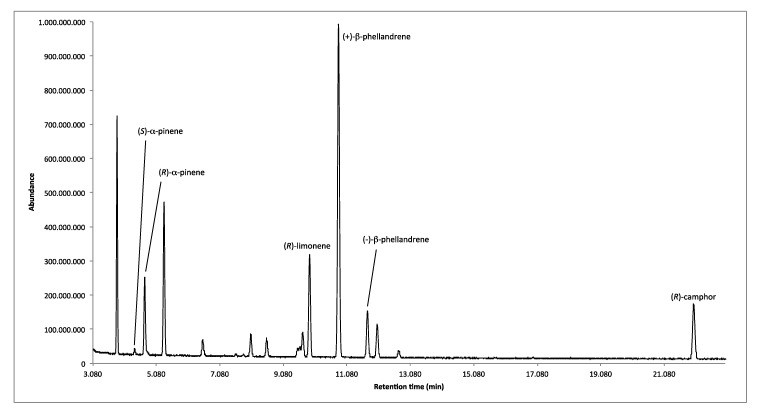
Enantioselective GC pattern of *L. heteromorpha* EO on diacethyl terbutylsilyl-β-cyclodextrin capillary column.

The recognition of chiral components in the EO was carried out with an enantioselective GC column, coated with 2,3-diacethyl-6-tert-butylsilyl-β-cyclodextrin as chiral selector. The enantiomers of α-pinene, β-phellandrene and the enantiomerically pure (*R*)-limonene and (*R*)-camphor were recognized. Additionally, *allo*-aromadendrene was probably present in two enantiomeric forms, however, its configuration was not attributed because of the lack of the reference standard of at least one enantiomer. The percentage enantiomeric distribution and enantiomeric excess (*ee*) of the identified enantiomers are shown in Table 2. Figure 1 reports the enantioselective GC pattern of *L. heteromorpha* EO.

#### 2.1.3. Characterization of (−)-Ledol (**1**)

(−)-Ledol (**1**) is the only sesquiterpene alcohol present in this EO, counting for more than 21% of the total analysis. Its molecular structure is shown in Supplementary Material. In order to set up the preparative purification method, four fractions of increasing polarity were previously obtained by normal phase preparative thin layer chromatography (TLC), indicated with numbers from 1 to 4. These fractions were analysed under the same conditions as those adopted for the GC-MS analysis of the total oil. From the GC-MS patterns of the resulting fractions, it can be observed that fraction 3 mainly consists of (−)-ledol (see Supplementary Material).

After column chromatography (CC) fractionation, a sample of (−)-ledol was obtained, whose molecular structure was confirmed by ^1^H and ^13^C NMR. The resulting spectroscopic data were identical to those reported in the literature. Also the specific rotatory power was consistent with the value of literature for (−)-ledol [25]. According to ^1^H NMR, the purity of compound (1) can be estimated of about 95%.

#### 2.1.4. Characterization of (−)-Caryophyllene Oxide (**2**)

During the purification process of compound (**1**), a second minor sesquiterpene was obtained as a by-product, and it was identified as caryophyllene oxide (**2**) by ^1^H and ^13^C NMR experiments [26]. Its amount and purity were not sufficient to measure reliably the specific rotatory power; however, it showed to be levorotatory. According to ^1^H NMR, the purity of compound (**2**) can be estimated of about 85%.

### 2.2. Ethyl Acetate Extract of Lepechinia Radula

The ethyl acetate extract of *L. radula* was investigated for secondary metabolites purification and characterization.

#### 2.2.1. Identification of Spathulenol (**3**)

Spathulenol (**3**) is a quite common sesquiterpene alcohol, obtained by normal phase fractionation of the ethyl acetate extract. The resulting white solid was identified as spathulenol by ^1^H NMR, electron impact mass spectrometry (EIMS), and GC(RI). LRI is referred to the homologous series of *n*-alkanes on a DB-5 column (LRI = 1583). All spectroscopic and gas chromatographic data were in agreement with those reported in the literature [27,28]. Neither ^13^C NMR nor specific rotatory power experiments were run because of the small amount obtained.

#### 2.2.2. Identification of Angustanoic Acid E (**4**)

Angustanoic acid E (**4**) was obtained as a crystalline compound by normal phase column chromatography, eluting with a mixture of hexane/ethyl acetate. Its purity was controlled by normal phase TLC, with hexane/ethyl acetate 90:10 as eluting phase (Rf: 0.71). The structure was elucidated by ^1^H, ^13^C and DEPT NMR experiments. All spectroscopic data were consistent with those reported in the literature [29].

#### 2.2.3. Identification of 5-hydroxy-4′,7-dimethoxy flavone (**5**)

A yellowish crystalline compound was isolated and identified as 5-hydroxy-4′,7-dimethoxy flavone (**5**) by ^1^H and ^13^C NMR experiments; the results were in perfect agreement with those reported in the literature [28]. Its purity was controlled by normal phase TLC with hexane/ethyl acetate 90:10 as eluting phase (Rf: 0.67).

### 2.3. Ethyl Acetate Extract of Lepechinia paniculata

The ethyl acetate extract of *L. paniculata* was studied for secondary metabolites purification and characterization.

#### 2.3.1. Identification of Ledol (**3**) and Guaiol (**6**)

Ledol (**3**) was identified in a fraction eluted with hexane/ethyl acetate 90:10, also containing guaiol (**6**). The fraction was analysed by GC(RI), where the two sesquiterpenoids were separated and identified by comparison of their LRIs and EIMS spectra to those of the literature [27] and to the reference sample isolated from the EO of *L. heteromorpha*.

#### 2.3.2. Characterization of (−)-Carnosol (**7**)

Carnosol was isolated from a more polar fraction (hexane/ethyl acetate about 80:20) as white crystals. The sample was submitted to ^1^H NMR providing spectral data identical to those obtained from a reference sample, isolated and characterized by X-ray diffraction from *L. mutica* [6].

## 3. Discussion

### 3.1. EO of L. Heteromorpha

In the EO of *L. heteromorpha*, 25 constituents were identified and quantified. Major components were viridiflorene (27.3%), (*E,E*)-α-farnesene (1.4%), ledol (21.2%), spirolepechinene and (*E*)-β-caryophyllene (7.1% each), *allo*-aromadendrene (6.1%), camphor (1.7%), limonene (1.3%) and β-phellandrene (4.6%). Sesquiterpenoids were the main fraction of the EO, accounting for about 85% of the whole sample. In particular, (−)-ledol is the only sesquiterpene alcohol in the EO. Its abundance (21.2%) makes this EO a good source of this compound in a seemingly pure enantiomeric form, easy to isolate by normal phase liquid chromatography. Enantioselective GC showed that only α-pinene and β-phellandrene were present with an enantiomeric excess, the others monoterpenes seemed to present as pure enantiomers. However, with the exception of (*R*)-camphor and (*R*)-limonene, it was not possible to attribute the absolute configuration to others enantiomers because of the unavailability of reference standards. Concerning caryophyllene oxide, it has sometimes been considered an artefact, produced by spontaneous oxidation and indicating the aging of the EO. However, in this case, we are inclined to consider it as an original component, as the fraction was properly stored and analysed soon after distillation.

In a previous work [7], some of the authors compared the major constituents of the EOs obtained from many *Lepechinia* spp. From this comparison, a common qualitative and quantitative profile did not emerge and an almost equal contribution from monoterpenes and sesquiterpenes was observed. The most common constituent was δ-3-carene, present in seven of 14 species, followed by (*E*)-β-caryophyllene that was observed in five. In this respect, *L. heteromorpha* EO did not contain δ-3-carene but showed the presence of abundant (*E*)-β-caryophyllene (about 7.1%). Other major constituents, described at least in two species, were borneol, β-phellandrene, 1,8-cineole, camphor, ledol, α-pinene and β-pinene. Except for borneol, 1,8-cineole and camphor, all the other compounds were detected in *L. heteromorpha*. A more accurate comparison was performed between the EOs from flowers and leaves of *L. mutica*, where important quantitative differences were observed [6]. In fact, while monoterpene fraction was prevalent in flowers (about 55.5% of the whole EO), sesquiterpenes were dominant in leaves (63.2%). However, in both cases a sesquiterpene alcohol was the relative major constituent, being eudesm-7(11)-en-4-ol the most abundant in flowers (13.0%) and shyobunol in leaves (10.8%). The situation was quite different for *L. heteromorpha*, where most of the compounds detected in *L. mutica* were absent. In fact, although its EO was mainly composed of sesquiterpenes (85.0%), it presented only a total of 26 detected metabolites, against about 80 compounds in *L. mutica* flowers and leaves.

### 3.2. Ethyl Acetate Extract of L. Radula and L. Paniculata

As for the EO of *L. heteromorpha*, the ethyl acetate extracts of *L. radula* and *L. paniculata* also contained compounds typical of this genus, i.e., 5-hydroxy-4′,7-dimethoxy flavone (**5**), (−)-carnosol (**7**), both described in *L. mutica* [6], and ledol (**1**), isolated from the EO of *L. heteromorpha*. Ledol and guaiol are diastereoisomers of viridiflorol, a sesquiterpene alcohol also present in *L. mutica* [6]. However, the most interesting component is probably (−)-carnosol, i.e., a well-known diterpenoid characterized by a variety of biological activities [30,31,32,33,34,35]. In particular, it is important for its antioxidant properties in rosemary (*Rosmarinus officinalis*) [36] and for a remarkable activity as butyrylcholinesterase (BChE) inhibitor in *L. mutica* [37].

Concerning (−)-spathulenol, it has been described sometimes to be an artefact, deriving from (−)-bicyclogermacrene. Asakawa et al. showed that the fresh extracts of *Dicranolejeunea yoshinagana* and others liverworts contained (−)-bicyclogermacrene, which completely disappeared after some months, with a contemporary increase of the amount of (−)-spathulenol. The same conversion took place in four days with pure (−)-bicyclogermacrene [38].

Sesquiterpenes, diterpenes and flavonoids, closely related to those observed in the ethyl acetate extracts of *L. radula* and *L. paniculata*, were also identified in other *Lepechinia spp*., together with pentacyclic triterpenes and other metabolites. *L. speciosa* [39], *L. caulescens* [40,41], *L. meyeni* [42], *L. hastata* [42], *L. chamaedryoides* [43], *L. bullata* [12,44] and *L. graveolens* [8] were particularly relevant for the presence of daucane sesquiterpenes and abietanic diterpenes. In particular, terpenes such as spathulenol, carnosic acid, carnosol and spirolepechinene, or flavonoids such as pinocembrin, 5-hydroxy-4′,7-dimethoxyflavone and luteolin-7-O-glucuronide were obtained and purified from these species.

## 4. Materials and Methods

### 4.1. General Information

The GC(RI) analyses were carried out with an Agilent Technology (Wilmington, DE, USA) 6890N gas chromatograph, coupled to an Agilent Technology single quadrupole mass spectrometer, series 5973 INERT. The ion source was an Electron Impact (EIMS) at 70 eV. Samples were injected with an automatic split/splitless injector, series 7683. The instrument was equipped with a DB-5MS (5% phenyl-methylpolysiloxane) Agilent 122-5532 capillary column (30 m × 0.25 mm × 0.25 μm of film thickness).

Semi-quantitative GC analyses were performed with the same GC-MS system, equipped with a flame ionization detector (FID) under the same analytical conditions.

The enantioselective GC analysis was carried out with an enantioselective capillary column, based on 30% diacethylterbutylsilyl-β-cyclodextrin in PS-086 (25 m × 0.25 mm × 0.25 μm, purchased from MEGA, Milan, Italy), installed in the same GC-MS system. Helium was used as carrier gas (Indura, Guayaquil, Ecuador).

For preparative column chromatography (CC), silica gel 60, particle size 0.063–0.200 mm (Merck KGaA, Darmstadt, Germany), was used as stationary phase. Normal phase thin layer chromatography (TLC) plates, with fluorescence indicator at 254 nm, were bought from Sigma-Aldrich (Saint Louis, MO, USA). After exposure to ultraviolet (UV) light (254 and 366 nm), the plates were revealed with a sulphuric acid/vanillin based reactive.

The organic solvents, used for CC and TLC, were purchased from Brenntag (Guayaquil, Ecuador) and carefully distilled before using. For GC applications and optical activity, the solvents were analytical grade from Sigma-Aldrich. The specific rotatory power was measured in an automatic polarimeter (Jinan Hanon Instruments Co. Ltd., Jinan, China) model MRC P810. The chiral standards for the enantioselective analysis were provided, as original samples, by one of the authors (C.B.)

All NMR analyses were performed with a Varian 400 MHz spectrometer (Walnut Creek, CA, USA), using deuterated solvent from Sigma-Aldrich. Electron Spray Ionization-Mass Spectrometry (ESI-MS) data were obtained by direct injection in a Bruker Amazon Speed ion trap mass spectrometer (Bruker, Billerica, MA, USA).

Molecular structures and spectroscopic data of compounds 1–5 and 7 are available as Supplementary Material.

### 4.2. Plant Material

The leaves of *L. heteromorpha* were collected in November 2017, in canton Saruguro, province of Loja (Ecuador), at coordinates 3°39’20.66” S and 79°15’20.43” W (2870 m above sea level).

The leaves of *L. radula* were collected in June 2015 in the canton Celica, province of Loja (Ecuador), at coordinates 4°4’35.29” S and 79°55’39.55” W (2493 m above sea level).

*L. paniculata* leaves were collected also in June 2015 at S. Lucas, in the canton Loja, province of Loja (Ecuador), at coordinates 3°43°33.79” S and 79°15’74.29” W (2578 m above sea level). A sample of each species was deposited at the herbarium of the Universidad Técnica Particular de Loja (UTPL), Ecuador, with voucher code HUTPL13744, PPN-LA-034 and PPN-LA-020, respectively. The samples were collected under governmental permission (MAE-DNB-CN-2016-0048) and identified, in the same university (UTPL), by two of the authors (N.C. and V.M.). The fresh leaves of *L. heteromorpha* were immediately distilled after collection, while *L. radula* and *L. paniculata* were dried in the dark at 35 °C before using.

### 4.3. EO of L. heteromorpha

#### 4.3.1. Distillation of the EO

Four samples of *L. heteromorpha* fresh leaves (1.50, 1.51, 1.63 and 1.50 kg, respectively) were distilled for 4 h in four stainless steel Clevenger-type apparatus. The distillates had to be recovered with a solvent because of their very high viscosity. Hence, 100 ml of dichloromethane, spiked with isopropyl hexanoate as internal standard, were added to the aqueous phase. The separated organic phase was then dried over anhydrous sodium sulphate for 30 min and directly analysed without solvent evaporation. The yield was calculated analytically, as a mean value of the four distillations.

#### 4.3.2. Qualitative and Semi-quantitative Analysis

Each sample was analysed by GC(RI), injecting 1 µl of the previously described EO solution in split mode (40:1), with the injector set at 220 °C. The following oven temperature program was used: 60 °C for 5 min, a gradient of 3 °C/min until 180 °C, then a gradient of 15 °C/min up to 250 °C, held for 5 min. The linear retention index (LRI) was calculated for each chromatographic peak, according to Van Den Dool and Kratz [45,46], using a mixture of the homologous series of *n*-alkanes, from C_9_ to C_20_. The mass spectrometer detector was set in SCAN mode, with 45–350 *m/z* range for the EO and 40–350 *m/z* for compounds purified after solvent extraction, with a 3 min solvent delay. The carrier gas was maintained at the constant flow of 1 ml/min. The EO components were identified by comparison of the respective EIMS spectra and LRIs to those stored in a reference library [25], with a LRI tolerance of +/− 10 units.

The semi-quantitative analysis was performed by GC-FID, with the same method and instrumental configuration described for GC(RI). The quantification was achieved by internal calibration, calculating the relative response factor (RRF) of each constituent, referred to the internal standard, on the basis of its combustion enthalpy [47,48,49]. Isopropyl hexanoate, instead of the methyl octanoate reported in the literature, was chosen as internal standard for this application. This approximation is justified by the principle that the RRF, for isomeric compounds analysed in FID, is about equal to 1 because the combustion enthalpy only depends on the molecular formula.

#### 4.3.3. Enantioselective GC Analysis

The enantioselective GC analysis was run with the following oven temperature program: 60 °C for 5 min, a gradient of 2 °C/min up to 220 °C, hold for 2 min. Injection and MS conditions were the same as those reported in the previous paragraph. The elution order of the separated enantiomers was determined by injection of enantiomerically pure standards, available in the laboratory of one of the authors (C.B.).

#### 4.3.4. Purification of (−)-Ledol and (−)-Caryophyllene oxide

A preliminary preparative normal phase TLC separation was carried out with 5 mg of the EO distilled from *L. heteromorpha*. The plate was eluted with a mixture of dichloromethane/methanol 95:5, the analytes detected under UV light (254 and 360 nm) and a small section of the same plate revealed with vanillin and 5% sulphuric acid. After scraping the separated bands and eluting them with ethyl acetate, the fractions were analysed by GC-MS, under the same analytical conditions described above (see Supplementary Material for chromatograms).

An amount of 1.4 g of the EO of *L. hereromorpha* was submitted to normal phase column chromatography (silica gel). A weight ratio of 1:100 between sample and stationary phase was applied. The elution was achieved isocratically, with a mixture of hexane/dichloromethane 40:60, collecting fractions of 10 mL. The pooled fraction containing sesquiterpenoids (469.2 mg) was again fractionated on normal phase CC to achieve a better purification of the major constituent, with a sample/stationary phase ratio of 1:200 by weight and an isocratic elution (hexane/dichloromethane 50:50). Fractions of 15 mL were collected, from where two compounds were isolated and identified: ledol (459.0 mg) and caryophyllene oxide (8.1 mg). The optical activity of both compounds was determined to be levorotatory.

### 4.4. Ethyl Acetate Extract Components of L. radula and L. paniculata Leaves

#### 4.4.1. Obtainment of Ethyl Acetate Extracts

Both species, *L. radula* and *L. paniculata*, were submitted to the same extraction process. 300 g of dry leaves were crushed and submitted to room temperature solvent extraction. The plant material was macerated in ethyl acetate for two hours; this operation was repeated three times with fresh solvent, to obtain an almost exhaustive extraction. The resulting solutions were pooled and the solvent removed by vacuum distillation at 30–35 °C, affording 20.2 g dry extract for *L. paniculata* and 12.1 g for *L. radula*.

#### 4.4.2. Chlorophyll Removal

Chlorophylls were removed from the extracts by selective absorption on C18 reversed phase. A vacuum funnel, equipped with a syntherized glass septum, was packed with C18 reversed phase suspended in pure methanol. The ratio sample/stationary phase was 1:10 by weight. After packing, the organic solvent was completely removed by washing with a great excess of a mixture CH_3_OH/H_2_O 98:2 for *L. paniculata* and 97:3 for *L. radula*. The extract (3.0 g) was then processed by dissolving it in a suitable volume of the same hydro-methanolic mixture used to condition the stationary phase. The whole volume was then eluted under vacuum over the funnel, obtaining a quantitative retention of chlorophylls. Finally, the stationary phase was washed with a large excess of pure methanol, followed by pure ethyl acetate. The resulting solutions were controlled by reversed phase TLC with pure methanol as eluent, to ensure the absence of compounds of interest retained with the chlorophylls.

#### 4.4.3. Preparative Isolation of Secondary Metabolites from *L. radula*

A normal phase CC was carried out with 1.0 g of chlorophyll-free ethyl acetate extract, over 100 g of silica gel. The sample was eluted with a mixture of hexane/ethyl acetate, according to an increasing eluotropic strength gradient, from 90:10 to pure ethyl acetate, collecting volumes of about 10 mL. Fractions with similar TLC composition were pooled in 18 different samples and evaporated under vacuum. Further normal phase chromatographic fractionation of these fractions, under similar elution conditions and stationary phase ratio, provided spathulenol (**3**, 1.5 mg), angustanoic acid E (**4**, 8.0 mg) and 5-hydroxy-4′,7-dimethoxy flavone (**5**, 5.1 mg).

#### 4.4.4. Fraction Purification and Secondary Metabolites Isolation from *L. paniculata*

A normal phase CC was performed with 1.6 g of chlorophyll-free ethyl acetate extract, over 160 g of silica gel. The sample was eluted with a mixture of hexane/ethyl acetate, according to an increasing polarity gradient, from 95:5 to pure ethyl acetate, collecting volumes of about 10 mL. A total of 297 fractions were obtained and then pooled into 18 fractions, according to their qualitative composition in TLC. Purified fraction 7 (3.5 mg) appeared to be a mixture, containing two inseparable major compounds that were identified as ledol (**3**) and guaiol (**6**) after GC(RI) analysis.

Fraction 6 (20.1 mg) was submitted to further isocratic CC fractionation with hexane/ethyl acetate 95:5, giving 15.3 mg of carnosol (**7**).

## 5. Conclusions

This is the first phytochemical study on the EO obtained from *L. heteromorpha*, and on the metabolic composition of the ethyl acetate extracts from *L. radula* and *L. paniculata*. The EO of *L. heteromorpha* was qualitatively and quantitatively characterized and mainly consisted of 27 compounds (including two undetermined), most of them being sesquiterpenoids (63.9%). A major sesquiterpene alcohol, (−)-ledol (21.2%), and a minor sesquiterpene epoxide, (−)-caryophyllene oxide (1.0%), were isolated and their structures confirmed by NMR spectroscopy. Other major components were viridiflorene (27.3%), (*E,E*)-α-farnesene (1.4%), ledol (21.2%), spirolepechinene and (*E*)-β-caryophyllene (7.1% each), *allo*-aromadendrene (6.1%), camphor (1.7%), limonene (1.3 %) and β-phellandrene (4.6%).

From the ethyl acetate extract of *L. radula* spathulenol, angustanoic acid E and 5-hydroxy-4′,7-dimethoxy flavone were purified; *L. paniculata* afforded ledol, guaiol and (−)-carnosol. All the compounds isolated from the ethyl acetate extracts were described here for the first time in the respective species. Guaiol and ledol, identified in the extract of *L. paniculata*, are also present, respectively, in *L. radula* and *L. heteromorpha* EO.

## Figures and Tables

**Table 1:** Chemical analysis of *L. heteromorpha* essential oil (EO) on a DB-5ms column.

Reference LRIs	Calculated LRIs	Compounds	% ^1^	σ
932	931	α-Pinene	1.2	0.71
946	946	Camphene	2.4	0.45
969	976	β-Pinene	0.4	0.18
988	988	Myrcene	0.4	0.22
1003	1003	Mentha-1(7),8-diene	0.4	0.23
1007	1005	*iso*-Sylvestrene	0.2	0.13
1020	1015	*p*-Cymene	0.5	0.26
1022	1017	*o*-Cymene	1.0	0.01
1024	1029	Limonene	1.3	0.41
1025	1029	β-Phellandrene	4.6	1.02
1141	1145	Camphor	1.7	0.46
1400	1404	Sibirene	3.2	0.32
1417	1411	(*E*)-β-Caryophyllene	7.1	0.77
1439	1439	Aromadendrene	1.0	0.18
1449	1445	Spirolepechinene	7.1	0.93
1458	1451	*allo*-Aromadendrene	6.1	1.00
1452	1453	α-Humulene	1.2	0.19
1496	1484	Valencene	1.6	0.23
-	1487	Undetermined (MW 204)	3.7	1.84
1492	1489	*cis*-β-Guaiene	0.3	0.16
1496	1497	Viridiflorene	27.3	1.80
1505	1503	(*E,E*)-α-Farnesene	1.4	0.41
-	1509	Undetermined (MW 204)	0.2	0.12
1511	1516	δ-Amorphene	1.0	0.98
1559	1549	Germacrene B	1.6	0.83
1592 ^2^	1593	Caryophyllene oxide	1.0	0.59
1602	1601	(−)-Ledol ^3^	21.2	4.32
Monoterpene hydrocarbons			12.4	
Oxygenated monoterpenes			1.7	
Sesquiterpene hydrocarbons			62.8	
Oxygenated sesquiterpenes			22.2	
Others			-	
Total identified			99.1	

^1^ % expressed as g/100g, ^2^ Linear Retention Indices (LRI) from [24], ^3^ Identification confirmed by ^1^H and ^13^C NMR.

**Table 2:** Enantioselective analysis of *L. heteromorpha* EO on diacethyl terbutylsilyl-β-cyclodextrin column.

LRIs	Enantiomers	Enantiomeric Distribution (%)	*ee* (%)
917	(*S*)-α-pinene	8.7	82.6
926	(*R*)-α-pinene	91.3	
1054	(*R*)-limonene	100.0	100.0
1072	(+)-β-phellandrene	88.3	76.6
1091	(−)-β-phellandrene	11.7	
1262	(*R*)-camphor	100.0	100.0

## Supplementary Materials

The following are available online at http://www.mdpi.com/2223-7747/8/1/1/s1, Figure S1: GC-MS comparison of fractions from a preparative TLC analysis of *L. heteromorpha* EO. The fractions represent compounds that can be separated by normal phase liquid chromatography. Figure S2: ^1^H NMR (400 MHz) spectrum of compound **1** in CDCl_3_. Figure S3: ^13^C NMR (100 MHz) spectrum of compound **1** in CDCl_3_. Figure S4: ^1^H NMR (400 MHz) spectrum of compound **2** in CDCl_3_. Figure S5: ^13^C NMR (100 MHz) spectrum of compound **2** in CDCl_3_. Figure S6: ^1^H NMR (400 MHz) spectrum of compound **3** in CDCl_3_. Figure S7: ^1^H NMR (400 MHz) spectrum of compound **4** in CDCl_3_. Figure S8: ^13^C NMR (100 MHz) spectrum of compound **4** in CDCl_3_. Figure S9: ^1^H NMR (400 MHz) spectrum of compound **5** in CDCl_3_. Figure S10: ^13^C NMR (100 MHz) spectrum of compound **5** in CDCl_3_ Figure S11: ^1^H NMR (400 MHz) spectrum of compound **7** in CDCl_3_. Figure S12: ^13^C NMR (100 MHz) spectrum of compound **7** in CDCl_3_. Table S1: ^1^H NMR spectroscopic data for compounds **1**-**5** and **7** (400 MHz, δ ppm, CDCl_3_), Table S2: 13C NMR spectroscopic data for compounds **1**, **2**, **4**, **5** and **7** (100 MHz, δ ppm, CDCl_3_).
