# Supplementary material for: Phytochemistry of Three Ecuadorian Lamiaceae: Lepechinia heteromorpha (Briq.) Epling, Lepechinia radula (Benth.) Epling and Lepechinia paniculata (Kunth) Epling
Source: Plants (Basel). 2018 Dec 20;8(1):1. doi: 10.3390/plants8010001 (PMC6358884; doi:10.3390/plants8010001)
Supplement: Supplementary file 1 [file plants-08-00001-s001.pdf]

Supplementary material

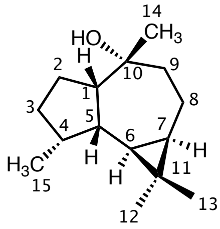
(1)

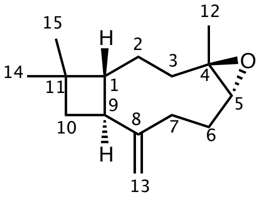
(2)

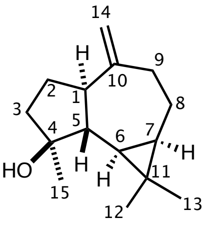
(3)

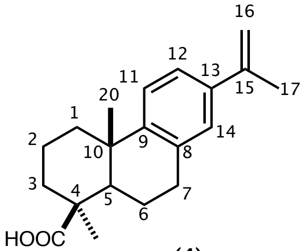
(4)

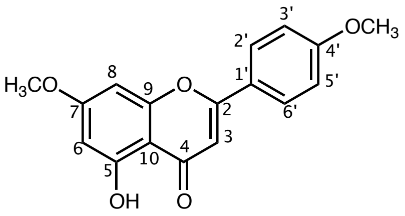
(5)

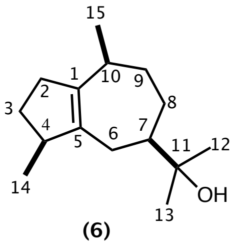
(6)

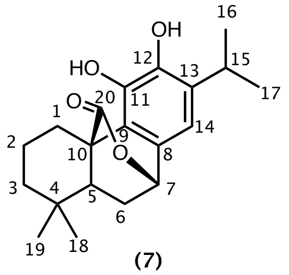
(7)

(-)-Ledol (**1**); $C_{15}H_{26}O$; waxy white powder. $[\alpha]_D^{20}$= -5.1 (c 1.0 in $CHCl_3$). EIMS *m/z* (%) = 222 (3, M), 204 (53), 189 (40), 162 (24), 161 (100), 149 (18), 148 (33), 147 (53), 136 (10), 135 (21), 134 (14), 133 (52), 123 (16), 122 (56), 121 (45), 120 (19), 119 (61), 117 (13), 115 (10), 111 (18), 109 (46), 108 (26), 107 (83) 106 (26), 105 (92), 96 (12), 95 (39), 94 (21), 93 (70), 92 (16), 91 (76), 83 (13), 82 (23), 81 (51), 80 (11), 79 (54), 77 (35), 69 (41), 67 (37), 65 (13), 55 (35), 53 (17). ^{1}H NMR: see Table S1. ^{13}C NMR: see Table S2.

(-)-Caryophyllene oxide (**2**); $C_{15}H_{24}O$; colourless liquid. EIMS *m/z* (%) = 220 (4, M), 205 (13), 202 (16), 187 (34), 177 (16), 174 (10), 173 (10), 164 (20), 163 (15), 162 (12), 161 (48), 159 (33), 149 (32), 147 (22), 146 (20), 145 (29), 138 (20), 137 (12), 136 (31), 135 (35), 134 (15), 133 (31), 132 (12), 131 (60), 129 (10), 123 (32), 122 (19), 121 (61), 120 (29), 119 (41), 118 (14), 117 (25), 115 (12), 111 (11), 110 (18), 109 (48), 108 (38), 107 (82), 107 (30), 105 (73), 97 (11), 96 (28), 95 (67), 94 (35), 93 (95), 92 (25), 91 (94), 84 (10), 83 (24), 82 (30), 81 (62), 80 (21), 79 (100), 78 (15), 77 (56), 71 (25), 70 (12), 69 (71), 68 (18), 67 (46), 65 (21), 55 (51), 53 (30). ^{1}H NMR: see Table S1. ^{13}C NMR: see Table S2.

Spathulenol (**3**), $C_{15}H_{24}O$; white powder. EIMS *m/z* (%) = 220 (15, M), 205 (61), 202 (26), 187 (26), 177 (17), 162 (39), 160 (12), 159 (43), 149 (19), 147 (33), 146 (24), 145 (21), 135 (16), 134 (15), 133 (22), 131 (23), 121 (23), 120 (17), 119 (57), 117 (17), 109 (14), 107 (36), 106 (25), 105 (40), 95 (23), 94 (13), 93 (48), 92 (13), 91 (50), 83 (10), 82 (19), 81 (23), 79 (35), 77 (19), 71 (21), 69 (35), 67 (28), 55 (27), 53 (15), 43 (100), 41 (63). ^{1}H NMR: see table 1.

Angustanoic acid E (**4**); $C_{22}H_{26}O_2$; white powder. ESI-MS *m/z* = 323 [M+1]. $[\alpha]_D^{20}$ = +102.3 (c 0.6 in $CHCl_3$). ^{1}H NMR: see Table S1. ^{13}C NMR: see Table S2.

5-hydroxy-4′,7-dimethoxy flavone (**5**); $C_{17}H_{14}O_5$; yellowish crystals. ESI-MS *m/z* = 299 [M+1]. ^{1}H NMR: see Table S1. ^{13}C NMR: see Table S2.

(-)-Carnosol (**7**); $C_{20}H_{26}O_4$; white crystals. ESI-MS *m/z* = 331 [M + 1]. $[\alpha]_D^{20}$ = -63.0 (c 0.75 in MeOH). ^{1}H NMR: see table S1. ^{13}C NMR: see table S2.

Table S1. ^{1}H NMR spectroscopic data for compounds **1-5** and **7** (400 MHz, δ ppm, $CDCl_3$).

Position	1	2	3	4	5	7
1	2.08 (1H, ddd, *J* = 9.2, 9.2, 6.4 Hz)	1.75 (1H, ddd, *J* = 10.3, 10.3, 4.2 Hz)	0.47 (1H, dd, *J* = 9.6, 11.6 Hz)	1.38 (1H, ddd, *J* = 13.2, 13.2, 4.0 Hz)	-	2.90 (1H, ddd, *J* = 13.0, 4.8, 1.6 Hz)
				2.28 (1H, m)	-	2.40 (1H, ddd, *J* = 13.0, 13.0, 4.4 Hz)
2	1.69 (1H, m)	1.45 (1H, m)	0.71 (1H, m)	1.62 (1H, m)	-	1.68 (1H, m)
	1.90 (1H, m)	1.64 (1H, m)		2.03 (1H, m)	-	
3	1.27 (1H, m)	2.10 (1H, m)	1.01 (1H, m)	1.09 (1H, ddd, *J* = 13.6, 13.6, 4.4 Hz)	6.57 (1H, s)	1.55 (1H, ddd, *J* = 13.4, 4.8, 1.6 Hz)
	1.71 (1H, m)	0.95 (1H, m)	1.96 (1H, m)	2.26 (1H, m)		1.28 (1H, ddd, *J* = 13.4, 13.4, 3.6 Hz)
4	1.98 (1H, m)	-	2.05 (1H, m)	-	-	-
		-	2.42 (1H, dd, *J* = 5.2, 13.6 Hz)	-	-	-
5	1.77 (1H, m)	2.85 (1H, dd, *J* = 10.6, 4.2 Hz)	-	1.57 (1H, dd, *J* = 12.0, 1.6 Hz)	-	1.73 (1H, dd, *J* = 10.8, 5.6 Hz)
			-		-	
6	0.33 (1H, dd, *J* = 9.8, 9.0 Hz)	1.35 (1H, m)	2.20 (1H, m)	2.19 (1H, dt, *J* = 6.0, 1.6 Hz)	6.36 (1H, d, *J* = 2.0 Hz) or	2.20 (1H, m)
		2.25 (1H, m)		2.06 (1H, m)	6.48 (1H, d, *J* = 2.0 Hz)	1.69 (1H, m)
7	0.71 (1H, ddd, *J* = 11.5, 9.0, 5.6 Hz)	2.10 (1H, m)	1.64 (1H, m)	2.80 (1H, m)	-	5.37 (1H, dd, *J* = 4.0, 1.6 Hz)
		2.35 (1H, m)	1.91 (1H, m)	2.92 (1H, dd, *J* = 16.8, 5.6 Hz)	-	
8	1.85 (2H, m)	-	1.54 (1H, m)	-	6.36 (1H, d, *J* = 2.0 Hz) or	-
		-	1.77 (1H, m)	-	6.48 (1H, d, *J* = 2.0 Hz)	-
9	1.71 (1H, m)	2.60 (1H, dd, *J* = 10.3, 8.5 Hz)	-	-	-	-
	1.90 (1H, m)	-	-	-	-	-
10	-	1.60 (1H, m)	1.31 (1H, m)	-	-	-
	-	1.69 (1H, m)		-	-	-
11	-	-	-	7.22 (1H, d, *J* = 8.0 Hz)	-	-
	-	-	-		-	-
12	0.93 (3H, d, *J* = 6.9 Hz)	1.20 (3H, s)	1.05 (3H, s)	7.24 (1H, d, *J* = 8.0 Hz)	-	-
					-	-
13	1.03 (3H, s)	4.86 (1H, d, *J* = 1.2 Hz)	1.04 (3H, s)	-	-	-

		4.97 (1H, d, *J* = 1.2 Hz)		-	-	-
14	0.98 (3H, s)	1.01 (3H, s)	4.66 (1H, d, *J* = 1.3 Hz)<br>4.68 (1H, d, *J* = 1.3 Hz)	7.14 (1H, bs)	-<br>-	6.64 (H, s)
15	1.14 (3H, s)	0.99 (3H, s)	1.28 (3H, s)	-<br>-	-<br>-	3.08 (1H, sept, *J* = 6.8 Hz)
16	-<br>-	-<br>-	-<br>-	5.32 (1H, bs)<br>5.02 (1H, bs)	-<br>-	1.22 (3H, d, *J* = 7.2 Hz) or<br>1.23 (3H, d, *J* = 7.2 Hz)
17	-<br>-	-<br>-	-<br>-	2.12 (3H, s)	-<br>-	1.22 (3H, d, *J* = 7.2 Hz) or<br>1.23 (3H, d, *J* = 7.2 Hz)
18	-<br>-	-<br>-	-<br>-	1.34 (3H, s)	-<br>-	0.90 (3H, s)
19	-<br>-	-<br>-	-<br>-	-<br>-	-<br>-	0.86 (3H, s)
20	-<br>-	-<br>-	-<br>-	1.12 (3H, s)	-<br>-	-<br>-
2' and 6'	-	-	-	-	7.01 (2H, d, *J* = 8.8 Hz) or 7.84 (2H, d, *J* = 8.8 Hz)	-
3' and 5'	-	-	-	-	7.01 (2H, d, *J* = 8.8 Hz) or 7.84 (2H, d, *J* = 8.8 Hz)	-
4'	-	-	-	-	-	-
-OCH_3	-	-	-	-	3.88 (3H, s, OCH_3)	-
-OCH_3	-	-	-	-	3.89 (3H, s, OCH_3)	-
-OH (12 or 20)	-	-	-	-	-	5.74 (1H, s)

Table S2. ^{13}C NMR spectroscopic data for compounds **1**, **2**, **4**, **5** and **7** (100 MHz, δ ppm, $CDCl_3$).

Position	1	2	4	5	7
1	53.9	50.9	39.4	-	29.3
2	24.8	27.3	20.0	164.2	19.0
3	31.7	39.3	37.5	104.3	41.1
4	38.6	60.0	44.0	182.5	34.7
5	40.9	63.9	52.9	162.7	45.6
6	23.6	29.9	21.0	98.2	29.9
7	25.1	30.0	32.2	165.6	78.0
8	20.4	152.0	135.2	92.8	132.2
9	39.4	48.9	147.5	157.8	121.7
10	74.7	39.9	38.6	105.7	48.5
11	19.3	34.2	125.6	-	141.9
12	15.5	17.1	123.2	-	141.2
13	28.8	112.9	138.4	-	133.0
14	30.7	21.8	126.2	-	112.4
15	16.1	30.3	143.1	-	27.4
16	-	-	111.8	-	22.6
17	-	-	21.9	-	22.7
18	-	-	28.8	-	31.8
19	-	-	183.1	-	19.8
20	-	-	23.2	-	176.1
1′	-	-	-	123.7	-
2′	-	-	-	128.2	-
3′	-	-	-	114.6	-
4′	-	-	-	162.3	-
5′	-	-	-	114.6	-
6′	-	-	-	128.2	-
7-OMe	-	-	-	55.9	-
4′-OMe	-	-	-	55.7	-

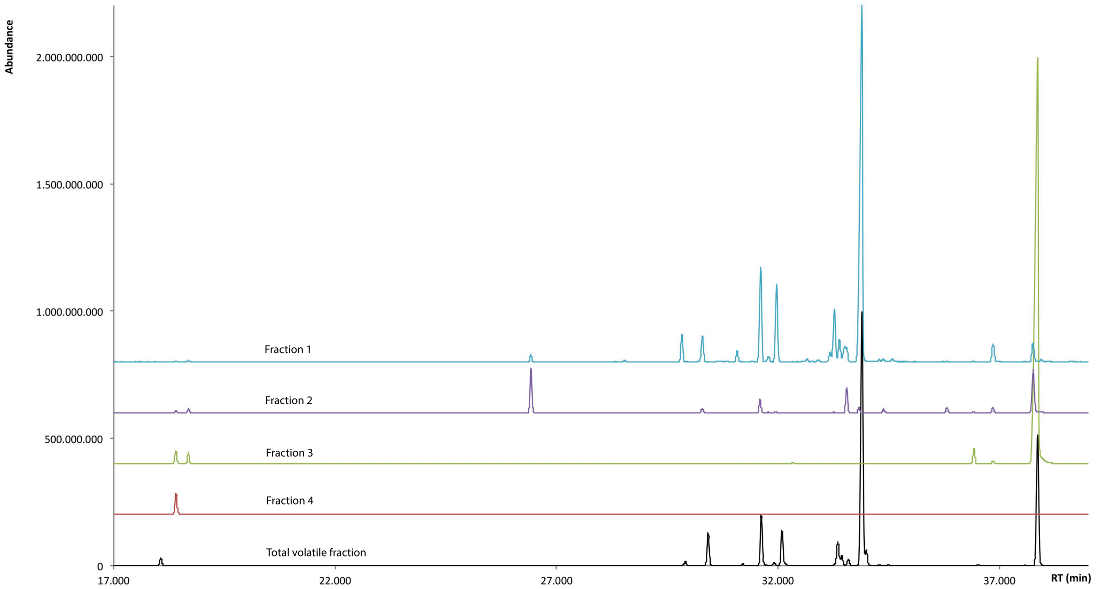

Figure S1. GC-MS comparison of fractions from a preparative TLC analysis of *L. heteromorpha* EO. The fractions represent compounds that can be separated by normal phase liquid chromatography.

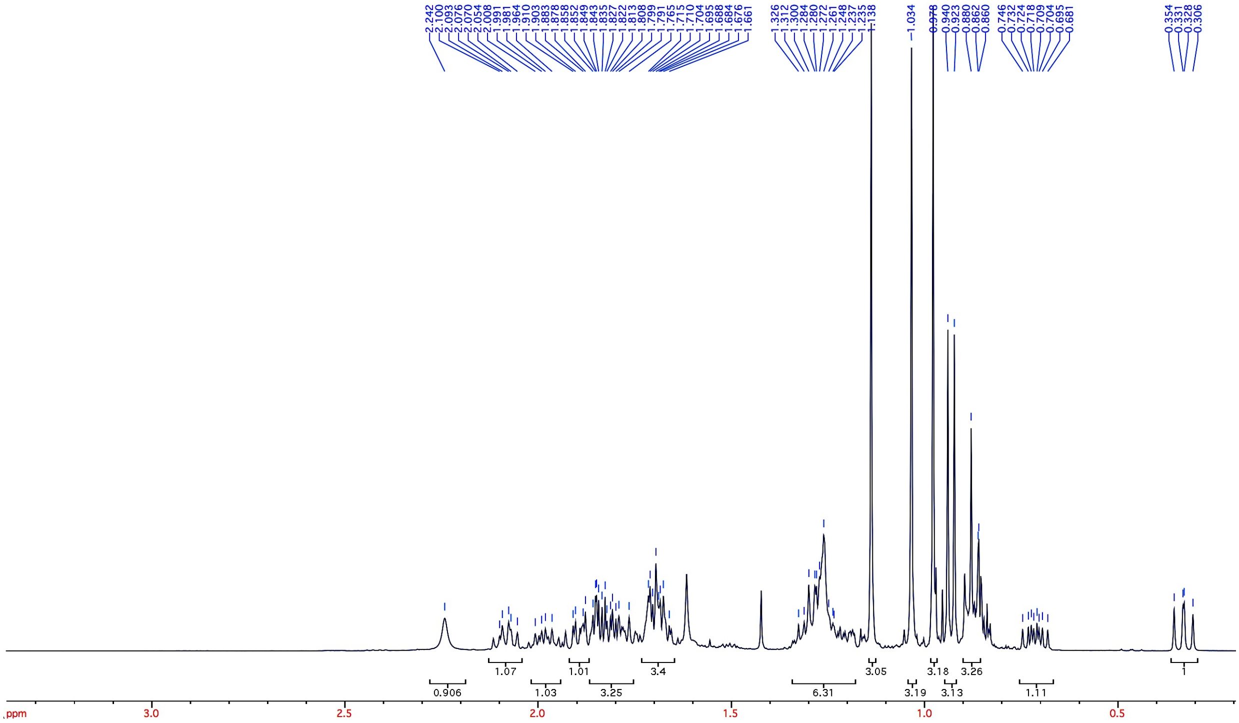

Figure S2. ^{1}H NMR (400 MHz) spectrum of compound (**1**) in $CDCl_3$

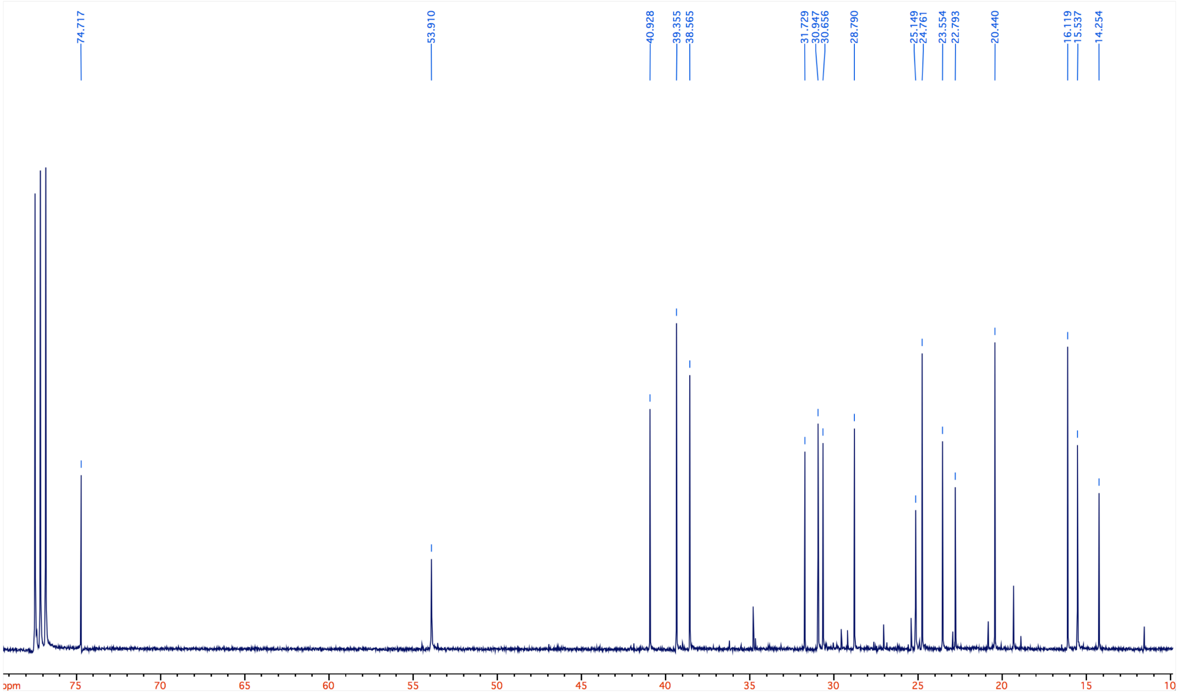

Figure S3. ^{13}C NMR (100 MHz) spectrum of compound (**1**) in $CDCl_3$

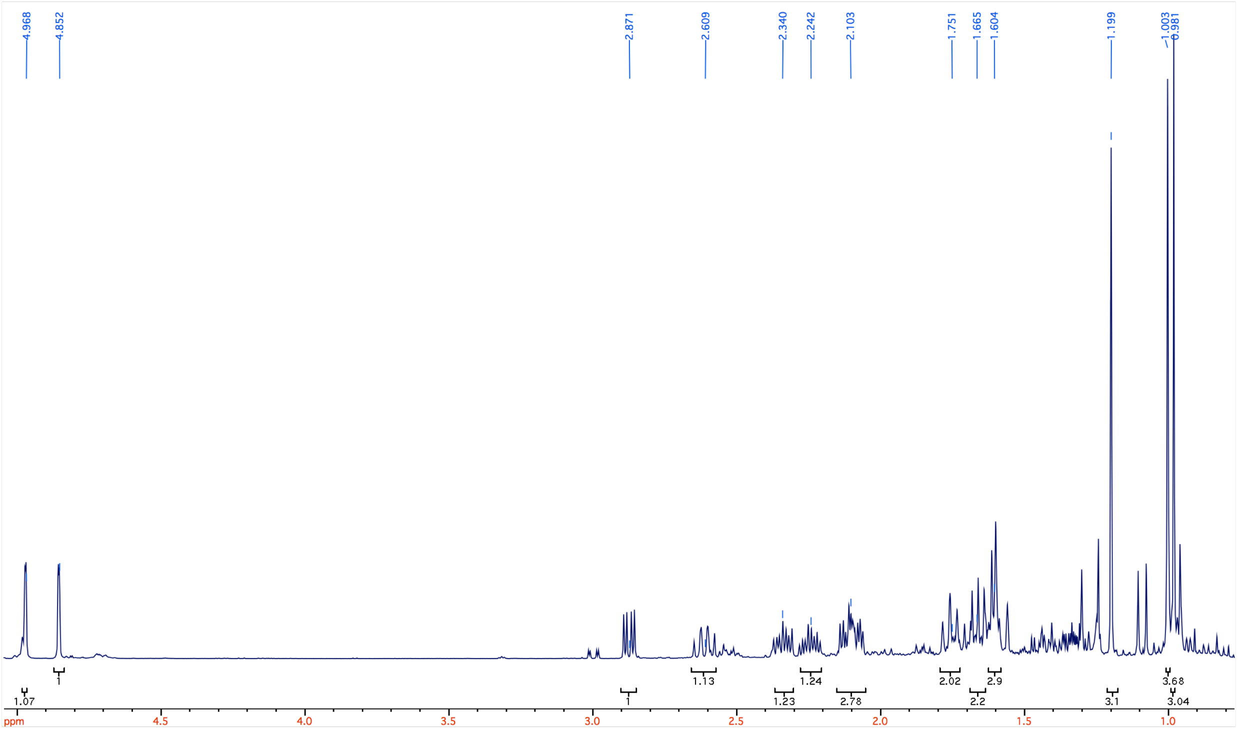

Figure S4. ^{1}H NMR (400 MHz) spectrum of compound (**2**) in $CDCl_3$

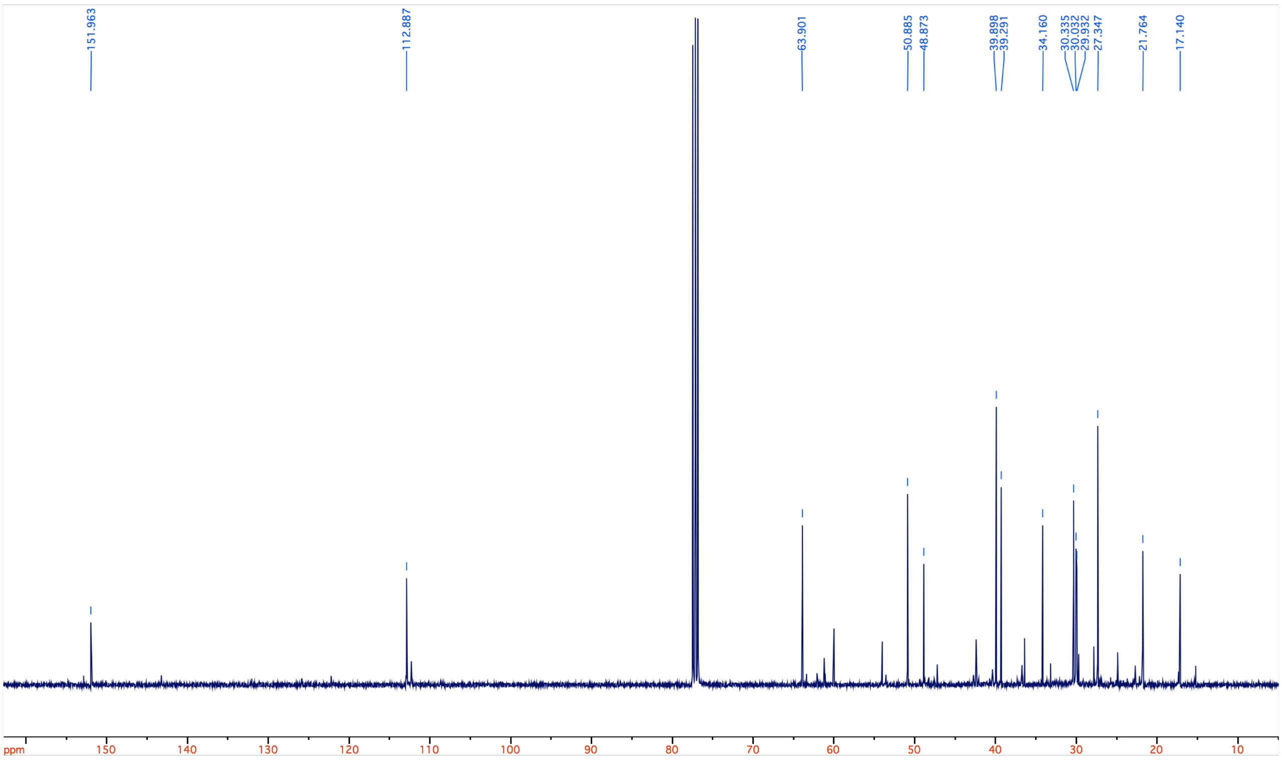

Figure S5. ^{13}C NMR (100 MHz) spectrum of compound (**2**) in $CDCl_3$

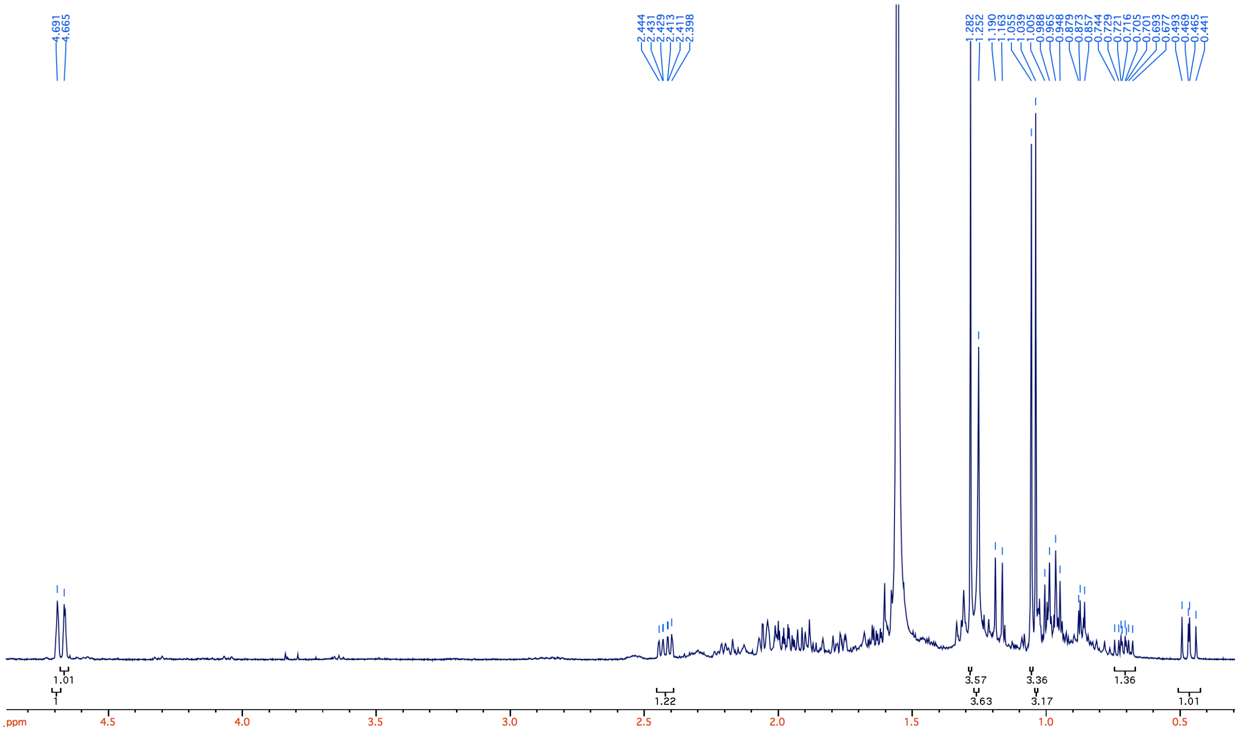

Figure S6. ^{1}H NMR (400 MHz) spectrum of compound (**3**) in $CDCl_3$

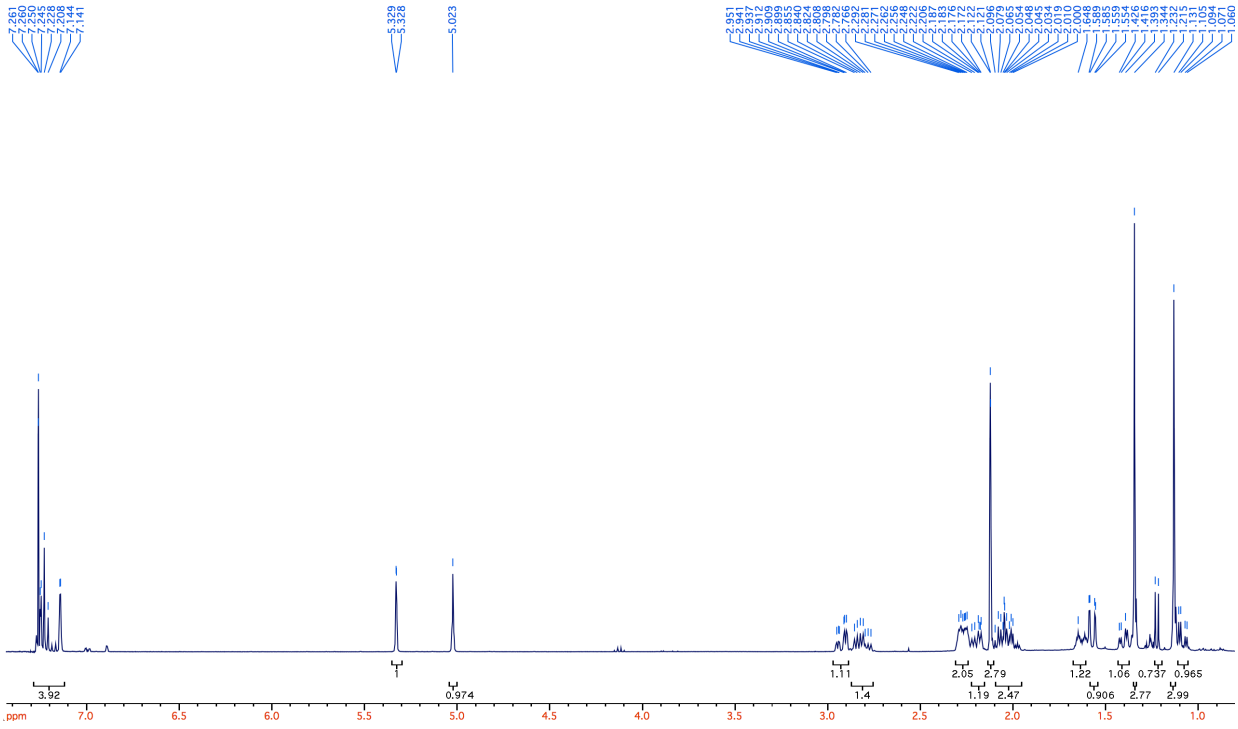

Figure S7. ^{1}H NMR (400 MHz) spectrum of compound (**4**) in $CDCl_3$

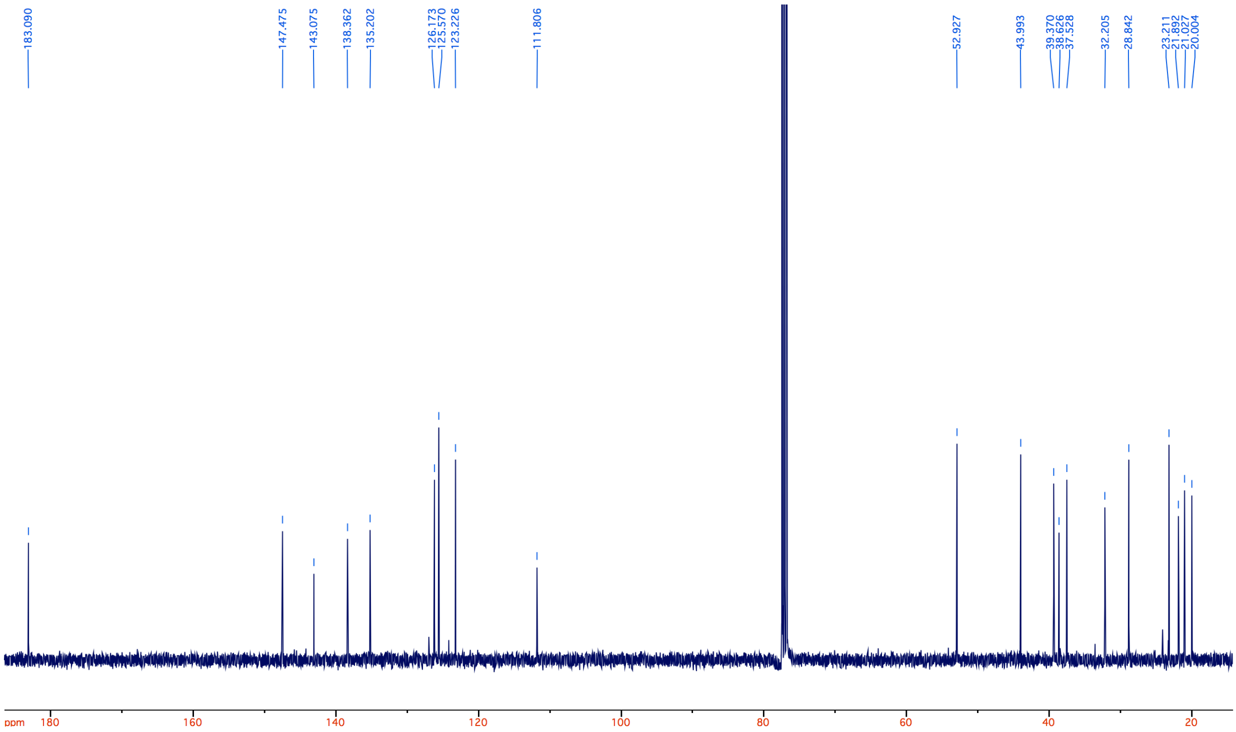

Figure S8. ^{13}C NMR (100 MHz) spectrum of compound (**4**) in $CDCl_3$

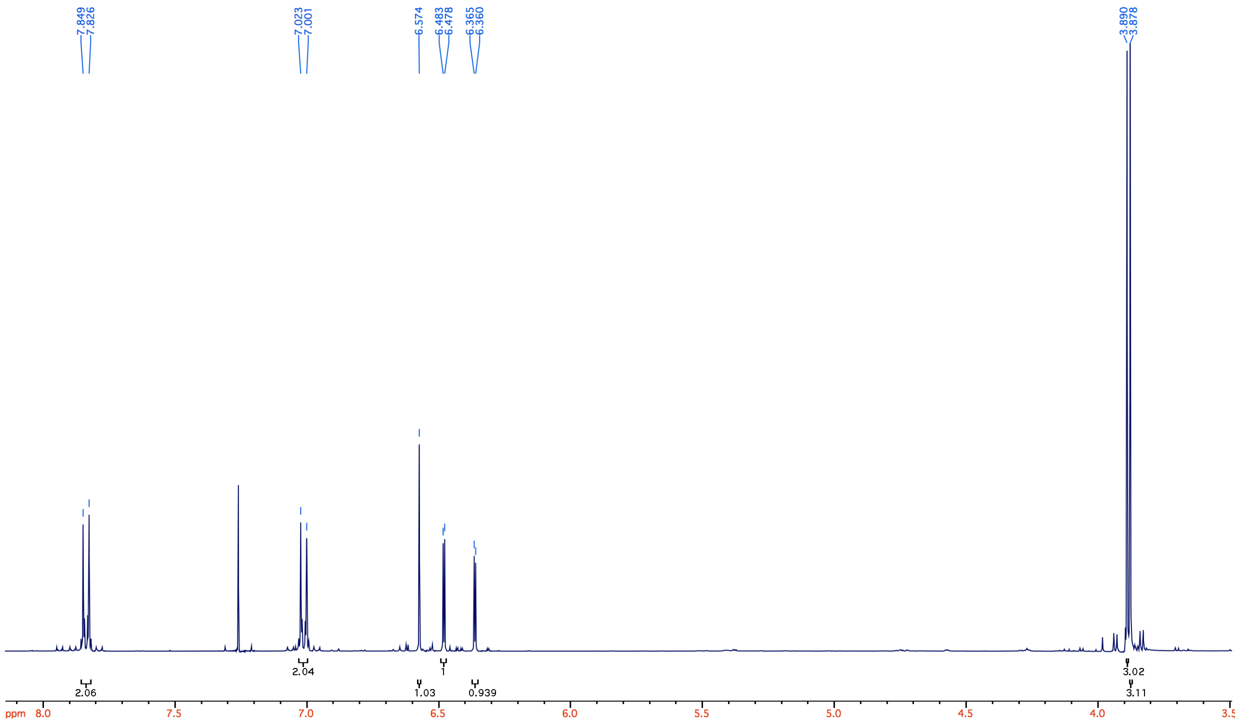

Figure S9. ^{1}H NMR (400 MHz) spectrum of compound (**5**) in $CDCl_3$

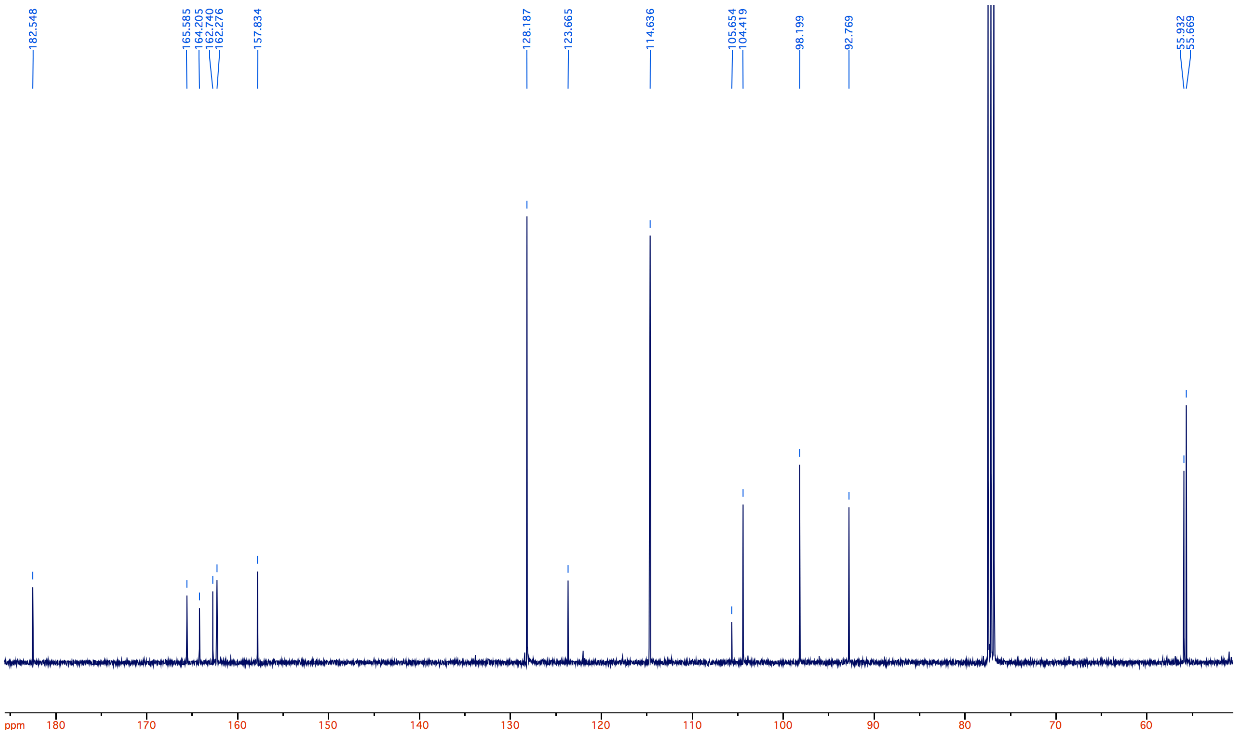

Figure S10. ^{13}C NMR (100 MHz) spectrum of compound (**5**) in $CDCl_3$

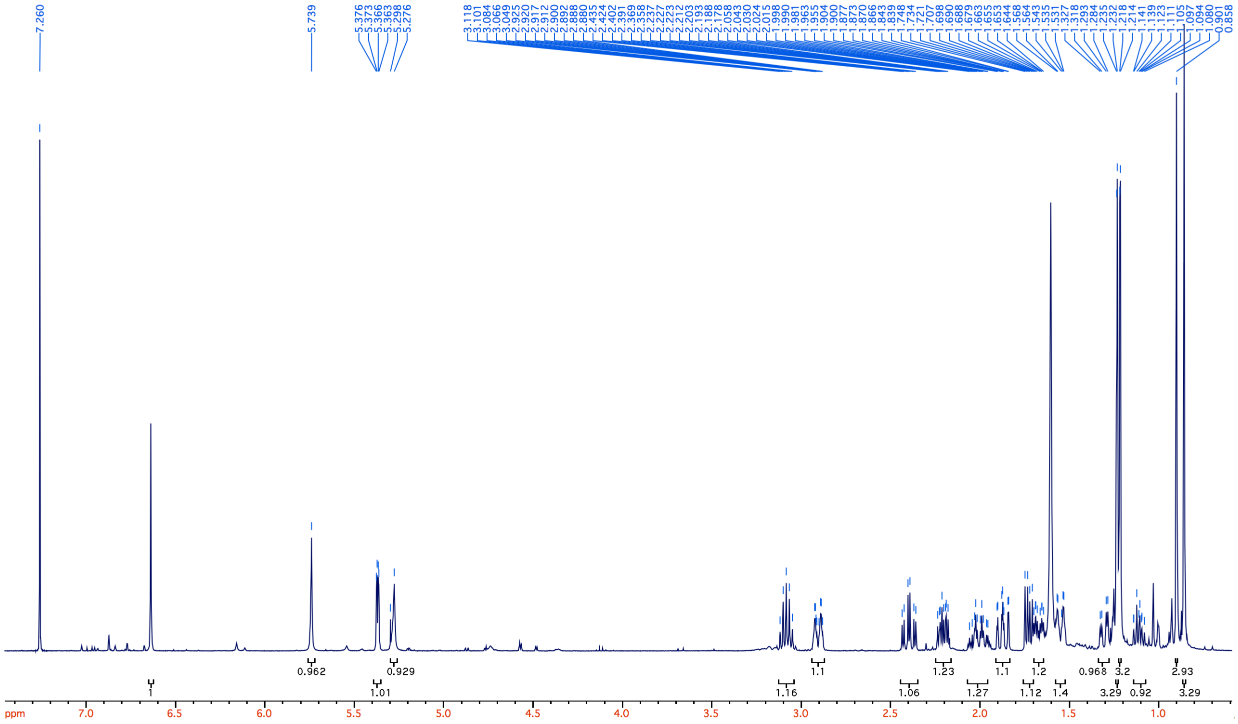

Figure S11. ^{1}H NMR (400 MHz) spectrum of compound (**7**) in $CDCl_3$

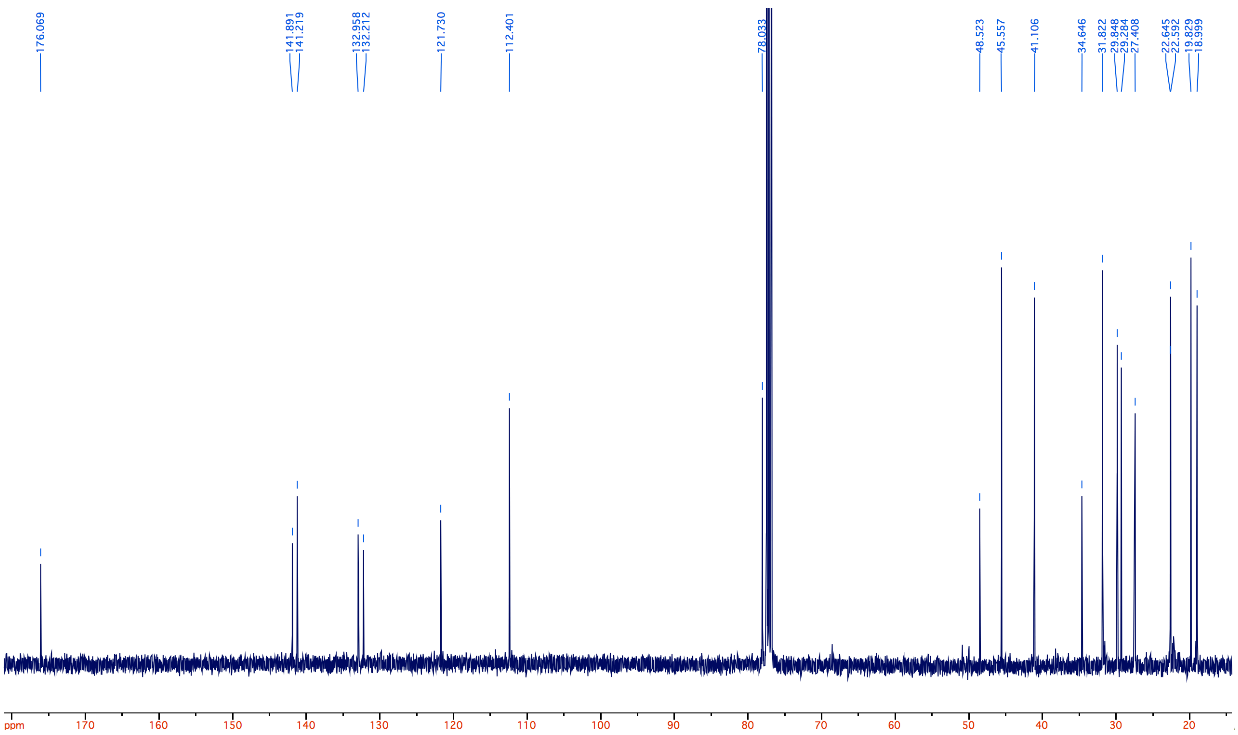

Figure S12. ^{13}C NMR (100 MHz) spectrum of compound (**7**) in $CDCl_3$
